# Identification of CHEK1, SLC26A4, c-KIT, TPO and TG as new biomarkers for human follicular thyroid carcinoma

**DOI:** 10.18632/oncotarget.10166

**Published:** 2016-06-18

**Authors:** Anne-Marie Makhlouf, Zhanna Chitikova, Marc Pusztaszeri, Margaret Berczy, Celine Delucinge-Vivier, Frederic Triponez, Patrick Meyer, Jacques Philippe, Charna Dibner

**Affiliations:** ^1^ Department of Medical Specialties, Faculty of Medicine, University of Geneva, Geneva, Switzerland; ^2^ Division of Clinical Pathology, University Hospital of Geneva, Geneva, Switzerland; ^3^ iGE3 Genomics Platform, University of Geneva, Geneva, Switzerland; ^4^ Department of Thoracic and Endocrine Surgery, University Hospital of Geneva, Geneva, Switzerland; ^5^ Division of Endocrinology, Diabetes, Hypertension and Nutrition, University Hospital of Geneva, Geneva, Switzerland

**Keywords:** follicular thyroid carcinoma, poorly differentiated thyroid carcinoma, NanoString analysis, diagnostic biomarkers

## Abstract

The search for preoperative biomarkers for thyroid malignancies, in particular for follicular thyroid carcinoma (FTC) diagnostics, is of utmost clinical importance. We thus aimed at screening for potential biomarker candidates for FTC. To evaluate dynamic alterations in molecular patterns as a function of thyroid malignancy progression, a comparative analysis was conducted in clinically distinct subgroups of FTC and poorly differentiated thyroid carcinoma (PDTC) nodules. NanoString analysis of FFPE samples was performed in 22 follicular adenomas, 56 FTC and 25 PDTC nodules, including oncocytic and non-oncocytic subgroups. The expression levels of *CHEK1, c-KIT, SLC26A4, TG* and *TPO* were significantly altered in all types of thyroid carcinomas. Based on collective changes of these biomarkers which correlating among each other, a predictive score has been established, allowing for discrimination between benign and FTC samples with high sensitivity and specificity. Additional transcripts related to thyroid function, cell cycle, circadian clock, and apoptosis regulation were altered in the more aggressive oncocytic subgroups only, with expression levels correlating with disease progression. Distinct molecular patterns were observed for oncocytic and non-oncocytic FTCs and PDTCs. A predictive score correlation coefficient based on collective alterations of identified here biomarkers might help to improve the preoperative diagnosis of FTC nodules.

## INTRODUCTION

Thyroid carcinomas are the most common type of endocrine malignancies, with an incidence steadily increasing worldwide [1]. The classification of thyroid carcinomas is made according to cell origin, with well-differentiated thyroid carcinomas (papillary thyroid carcinoma (PTC) and follicular thyroid carcinoma (FTC)) being the most frequent types. FTC is further sub-classified into oncocytic and non-oncocytic subtypes with distinct genomic, epigenomic, proteomic and clinical profiles, according to the Cancer Genome Atlas Research Network. Poorly differentiated and undifferentiated (anaplastic) thyroid carcinomas (PDTC and ATC) are less common but more aggressive [2]. Fine-needle aspiration (FNA) biopsy is the standard diagnostic test recommended for the clinical evaluation of thyroid non-secreting nodules ≥ 1 cm [3]. While FNA allows for the reliable recognition of most classical PTC cases, it stays indeterminate in about 20–30% of cases, mostly for malignant follicular lesions. The differentiation between benign follicular adenoma and FTC is virtually impossible based on cytological features, since the hallmark of malignancy in FTC is the presence of capsular or vascular invasion, which cannot be assessed by FNA. Therefore, surgery is generally recommended for these patients [3]. Postoperatively, the majority of indeterminate cases are classified as benign, revealing a significant rate of unnecessary surgeries, complications, and even morbidity [1].

Numerous studies have aimed to find predictive factors of malignancy before patients undergo surgery, including genetic analyses and search for molecular biomarkers [4]. A particular effort has been thus undertaken in the field to explore molecular alterations and genetic mutations, which may allow for the accurate pre-operative clinical diagnostics of FTC ([5] and references therein). The presence of *RAS* point mutations or *PAX8/PPARγ* rearrangement in FTC might represent such diagnostic markers, with *RAS* demonstrating also a strong association with disease aggressiveness [6]. However, the sensitivity of these gene analyses is very low. Moreover, *RAS* or *PAX8/PPARγ* alterations are also found in a subset of follicular adenomas, therefore limiting their predictive value. Substantial efforts including large-scale screening studies have revealed numerous potential biomarkers for the preoperative diagnostic of FTC, however, none of those provide conclusive results for patients with indeterminate thyroid FNA cytology [7]. Therefore, the search for reliable preoperative markers of FTC cases with indeterminate cytology stays of utmost clinical importance.

Our recent work has allowed for the identification of new potential biomarkers for postoperative PTC FFPE samples [8] employing NanoString analysis [9]. Parallel assessment of changes in the expression levels of several biomarkers in the same sample has let us to establish a predictive score based on the combined changes of these candidate genes, and thus provides a more accurate diagnostic test compared to alterations of one transcript only. Moreover, cell cycle regulator *CHEK1* and circadian clock component *BMAL1* have been identified as potential biomarkers for PTC [8]. Employing the settings developed by us for the analysis of FFPE samples by NanoString [8], we now aimed at screening for potential biomarker candidates and molecular patterns in FTC, including different subgroups within the FTC category. To evaluate the dynamics of thus identified transcript alterations as a function of thyroid malignancy progression, the same analysis was conducted in PDTC nodules.

## RESULTS

### Transcriptional alterations in different subgroups of FTC nodules assessed by NanoString analysis

12 healthy thyroid samples and 22 benign thyroid nodules (follicular adenomas) obtained during planned thyroid surgeries (see Supplementary Table S1 for donor characteristics), were subjected to NanoString analysis. The panel of 22 genes (Supplementary Table S4), comprising those related to thyroid function, core clock, cell cycle and apoptosis, was analyzed (for gene selection details see Materials and Methods). NanoString analysis revealed that transcriptional pattern of the benign thyroid nodule was not significantly altered for any of 22 analyzed genes, in comparison to healthy thyroid tissue, with no transcript exhibiting significant difference in their false discovery rate (FDR) 5% (Supplementary Table S5).

In an attempt to identify the transcripts with altered expression levels upon FTC development, FTC samples (see Supplementary Table S2 for donor characteristics and postoperative diagnosis) were analyzed by NanoString. Expression levels of the same 22 genes (Supplementary Table S4) were compared between 56 FTC and 22 follicular adenomas described above. NanoString analysis revealed that the cell-cycle related transcript *CHEK1* was upregulated 9-fold in FTC, compared to the benign counterpart (Table 1, subgroup 1). Along with the upregulation of *CHEK1*, a 2 to 5-fold downregulation of *c-KIT, SLC26A4, TG* and *TPO* transcript levels was observed in FTC (Table 1, subgroup 1).

**Table 1:** Altered transcript expression in FTC samples as compared to benign counterparts

Gene	*P*-value (FTC vs benign)	*P*-value with FDR (FTC vs benign)	Fold change	Total Number of samples FTC/benign	Number of samples with expression value > 50 (linear scale) FTC/benign
*1. Without consideration of FTC type*					
*CHEK1*	0.0000	0.0001	9.06	56/22	49/10
*c-KIT*	0.0032	0.0091	−5.06	56/22	48/22
*SLC26A4*	0.0028	0.0091	−2.19	56/22	56/22
*TG*	0.0001	0.0004	−2.43	56/22	56/22
*TPO*	0.0000	0.0001	−3.52	56/22	56/22
*2. Non-oncocytic FTC*					
*CHEK1*	0.0002	0.0044	7.74	32/22	27/10
*TPO*	0.0006	0.0062	−2.37	32/22	32/22
*3. Non-oncocytic FTC with vascular invasion*					
*CHEK1*	0.0002	0.0024	10.24	18/22	16/10
*TPO*	0.0001	0.0017	−3.14	18/22	18/22
*4. Oncocytic FTC*					
*CHEK1*	0.0001	0.0003	10.45	24/22	22/10
*c-KIT*	0.0001	0.0004	−10.13	24/22	19/22
*PER2*	0.0000	0.0000	−2.18	24/22	24/22
*SLC26A4*	0.0025	0.0054	−2.53	24/22	24/22
*SLC5A5*	0.0078	0.0157	−7.97	24/22	20/19
*TG*	0.0000	0.0000	−4.17	24/22	24/22
*TPO*	0.0000	0.0000	−5.42	24/22	24/22
*5. Oncocytic FTC without vascular invasion*					
*CHEK1*	0.0035	0.0130	8.14	11/22	10/10
*TG*	0.0001	0.0022	−2.75	11/22	11/22
*TPO*	0.0006	0.0063	−3.15	11/22	11/22
*6. Oncocytic FTC with vascular invasion*					
*BCL2*	0.0000	0.0000	−2.01	13/22	13/22
*CHEK1*	0.0002	0.0006	13.40	13/22	12/10
*FZD1*	0.0008	0.0019	−2.23	13/22	13/22
*c-KIT*	0.0000	0.0000	−25.54	13/22	9/22
*PER2*	0.0000	0.0000	−2.90	13/22	13/22
*SLC26A4*	0.0002	0.0006	−3.87	13/22	13/22
*SLC5A5*	0.0024	0.0045	−16.74	24/22	9/19
*TG*	0.0000	0.0000	−6.33	13/22	13/22
*TPO*	0.0000	0.0000	−9.31	13/22	13/22

To address a possible correlation between the oncocytic feature of FTC nodules, representing a more aggressive form of the disease, and clinical diagnostics we next compared transcript changes in non-oncocytic versus oncocytic subgroups. Furthermore, taking into account the clinical and molecular heterogeneity of FTC depending on the presence of capsular or vascular invasion, differential analysis of samples with and without capsular and vascular invasion was performed within each subgroup (Supplementary Table S2). As presented at Table 1 subgroup 2, FTC with non-oncocytic diagnostics and without capsular invasion exhibited a 7.7-fold upregulation of *CHEK1* and a 2-fold downregulation of *TPO*. Similar results, with slightly stronger fold changes for both markers, were observed for non-oncocytic FTC with vascular invasion (Table 1, subgroup 3). In the oncocytic subgroup of FTC (Table 1, subgroup 4), in addition to the 10-fold upregulation of *CHEK1* and the 5-fold downregulation of *TPO*, *c-KIT, SLC26A4, SLC5A5, TG* and *PER2* levels were downregulated. Strikingly, oncocytic samples without vascular invasion exhibited relatively milder changes in *CHEK1*, *TPO* and *TG* (Table 1, subgroup 5). The strongest alterations of *CHEK1, TPO*, *c-KIT, SLC26A4, SLC5A5, TG* and *PER2* were observed in FTC oncocytic samples with vascular invasion (Table 1, subgroup 6). Furthermore, two additional transcripts, *BCL2* and *FZD1*, were significantly downregulated in these samples. No significant differences in the molecular pattern were observed comparing subgroups with and without capsular invasion (data not shown).

### NanoString analysis of PDTC nodules

To address gene expression changes upon thyroid follicular carcinoma development, we next analyzed a group of 25 FFPE samples with postoperative PDTC diagnosis (Supplementary Table S3). NanoString analysis of 22 transcripts (Supplementary Table S4) was performed for these samples and compared to benign and FTC counterparts analyzed in parallel. The expression levels of *CHEK1*, *c-KIT, SLC26A4, TG* and *TPO* were altered in a more extreme manner in PDTC than in FTC (compare subgroups 1 in Tables 1, 2). In addition, the levels of *DIO2, KDR, CDKN1B, FZD1, BCL2, PER2, CRY2* and *SLC5A5* were strongly downregulated in PDTC. Next, transcript level changes in the oncocytic and non-oncocytic PDTC were evaluated separately. Consistent with the trend observed in FTC, relatively milder alterations were observed in the non-oncocytic PDTC subgroup for *CHEK1, DIO2, KDR, SLC26A4, SLC5A5, TG* and *TPO* (Table 2, subgroup 2). By contrast, along with *CHEK1* exhibiting 33-fold up-regulation and *c-KIT, SLC26A4, SLC5A5, TG,* and *TPO* exhibiting over 35-fold downregulation, the levels of *CDKN1B, DIO2, FZD*, and *KDR* were significantly down-regulated (Table 2, subgroup 3). Apoptosis marker *BCL2* and circadian core-clock genes *CRY2* and *PER2* were further downregulated in this subgroup if compared to all PDTCs (Table 2, compare subgroup 3 to 1).

**Table 2:** Altered transcript expression in PDTC samples as compared to benign counterparts

Gene	*P*-value (PDTC vs benign)	*P*-value with FDR (PDTC vs benign)	Fold change	Total number of samples PDTC/benign	Number of samples with expression value > 50 (linear scale) PDTC/benign
*1. Without consideration of PDTC type*					
*BCL2*	0.0000	0.0000	−2.63	25/22	25/22
*CDKN1B*	0.0093	0.0158	−3.68	25/22	24/22
*CHEK1*	0.0000	0.0000	25.14	25/22	25/10
*CRY2*	0.0000	0.0000	−2.07	25/22	25/22
*DIO2*	0.0001	0.0001	−3.10	25/22	25/22
*FZD1*	0.0000	0.0001	−2.68	25/22	25/22
*KDR*	0.0000	0.0000	−3.68	25/22	25/22
*c-KIT*	0.0002	0.0004	−9.98	25/22	19/22
*PER2*	0.0000	0.0000	−3.06	25/22	25/22
*SLC26A4*	0.0000	0.0000	−17.79	25/22	22/22
*SLC5A5*	0.0000	0.0000	−29.94	25/22	16/19
*TG*	0.0000	0.0000	−10.60	25/22	24/22
*TPO*	0.0000	0.0000	−28.68	25/22	23/22
*2. Non-oncocytic PDTC*					
*CHEK1*	0.0000	0.0000	20.96	15/22	15/10
*DIO2*	0.0086	0.0188	−2.29	15/22	15/22
*KDR*	0.0000	0.0000	−2.69	15/22	15/22
*SLC26A4*	0.0001	0.0005	−10.99	15/22	14/22
*SLC5A5*	0.0003	0.0011	−23.77	15/22	10/19
*TG*	0.0002	0.0009	−4.58	15/22	15/22
*TPO*	0.0000	0.0000	−17.02	15/22	14/22
*3. Oncocytic PDTC*					
*BCL2*	0.0000	0.0000	−5.37	10/22	10/22
*CDKN1B*	0.0057	0.0096	−5.97	10/22	9/22
*CHEK1*	0.0000	0.0000	33.02	10/22	10/10
*CRY2*	0.0000	0.0000	−3.38	10/22	10/22
*DIO2*	0.0000	0.0000	−4.87	10/22	10/22
*FZD1*	0.0000	0.0000	−5.53	10/22	10/22
*KDR*	0.0000	0.0000	−5.90	10/22	10/22
*c-KIT*	0.0000	0.0000	−43.36	10/22	6/22
*PER2*	0.0000	0.0000	−6.46	10/22	10/22
*SLC26A4*	0.0000	0.0000	−36.62	10/22	8/22
*SLC5A5*	0.0002	0.0003	−42.32	10/22	6/19
*TG*	0.0000	0.0000	−37.29	10/22	9/22
*TPO*	0.0000	0.0000	−62.72	10/22	9/22

### Alterations of CHEK1, c-KIT, SLC26A4, TG and TPO expression levels in FTC exhibit pair-wise correlations

Given that the NanoString approach allows for the assessment of numerous transcript levels within the same sample, we next performed pair-wise correlation analysis among the transcripts that exhibited the most pronounced alterations in FTC. Pair-wise correlation analyses of the combined set of 56 FTC samples enrolled in this study (Supplementary Table S2) revealed that alterations of *CHEK1, c-KIT, SLC26A4, TG* and *TPO* were significantly correlated (Table 3). Specifically, *c-KIT, SLC26A4, TG* and *TPO* exhibited moderate to strong positive correlations, while *CHEK1* was weakly inversely correlated with rest of the transcripts (Table 3). Therefore, this group of transcripts represents a plausible cluster of biomarkers whose collective changes are associated with FTC development, and might thus be potentially predictive of FTC diagnosis.

**Table 3:** Pair-wise correlation coefficients (R) for CHEK1, c-KIT, SLC26A4, TPO and TGlevels in FTCs

Genes	R	*p*-value
*CHEK1/c-KIT*	−0.19	0.0926
*CHEK1/SLC26A4*	−0.32	0.0045
*CHEK1/TG*	−0.51	0
*CHEK1/TPO*	−0.43	0.0001
*KIT/SLC26A4*	0.64	0
*KIT/TG*	0.45	0
*KIT/TPO*	0.63	0
*SLC26A4/TG*	0.64	0
*SLC26A4/TPO*	0.62	0
*TG/TPO*	0.79	0

### RAS mutation analysis in the FTC samples

To acquire additional valuable characteristics of the FTC nodules analyzed in this study by NanoString, we conducted *N-RAS61* and *H-RAS61* mutation analysis [10] for the same FTC nodules. As listed in Table 4, 8.9% (5/56) of FTC samples exhibited the *N-RAS61* mutation, in an agreement with previous reports [11]. A similar frequency of the *N-RAS61* mutation (8.3%; 2/24) was observed for the oncocytic FTC subgroup. Surprisingly, one out of 22 benign samples exhibited an *N-RAS61* mutation different from those detected in FTCs (Table 4). Of note, this particular type of *N-RAS61* mutation (Ala59Pro (c.175G > C)) detected in the benign sample has never been described previously. With regard to *H-RAS61,* one sample was identified as positive within all FTC samples. This sample was classified in the oncocytic FTC group (Table 4). No *H-RAS61* mutation was detected in the benign samples.

**Table 4:** NRAS61 and HRAS61mutation analysis

Group	Case	N-RAS	H-RAS
Benign samples (Supplementary Table S1)	1	WT	WT
	2	WT	WT
	3	WT	WT
	4	WT	WT
	5	WT	WT
	6	WT	WT
	7	WT	WT
	8	WT	WT
	9	WT	WT
	10	WT	WT
	11	WT	WT
	12	WT	WT
	13	Ala59Pro (c.175G>C)	WT
	14	WT	WT
	15	WT	WT
	16	WT	WT
	17	WT	WT
	18	WT	WT
	19	WT	WT
	20	WT	WT
	21	WT	WT
	22	WT	WT
FTC samples (Supplementary Table S2)	1^V^,^C^	WT	WT
	2^O^,^V^,^C^	WT	WT
	3^O^,^C^	WT	WT
	4^O^,^V^,^C^	WT	WT
	5^V^,^C^	WT	WT
	6^C^	WT	WT
	7^V^,^C^	Gln61Arg (c.182A>G)	WT
	8^V^,^C^	WT	WT
	9^C^	WT	WT
	10^C^	WT	WT
	11^O^,^V^	WT	WT
	12^V^	WT	WT
	13^V^	WT	WT
	14^C^	WT	WT
	15^O^,^V^,^C^	WT	WT
	16^C^	WT	WT
	17^C^	WT	WT
	18^V^	WT	WT
	19^C^	WT	WT
	20^V^,^C^	WT	WT
FTC samples (Supplementary Table 2)	21^V^	WT	WT
	22^V^	WT	WT
	23^V^	WT	WT
	24^O^,^C^	WT	WT
	25^O^,^C^	WT	WT
	26^V^	WT	WT
	27^O^,^C^	WT	WT
	28^O^,^V^,^C^	WT	WT
	29^O^,^C^	Gln61Arg (c.182A>G)	WT
	30^O^,^V^,^C^	WT	WT
	31^O^,^V^,^C^	WT	Gln61Arg (c.182A>G)
	32^O^,^C^	WT	WT
	33^O^,^V^,^C^	WT	WT
	34^O^,^V^,^C^	WT	WT
	35^O^,^V^,^C^	WT	WT
	36^O^,^C^	WT	WT
	37^O^,^C^	WT	WT
	38^O^,^C^	WT	WT
	39^O^,^V^,^C^	WT	WT
	40^V^	WT	WT
	41^C^	WT	WT
	42^C^	WT	WT
	43^V^,^C^	Gln61Arg (c.182A>G)	WT
	44^V^,^C^	WT	WT
	45^C^	WT	WT
	46^C^	WT	WT
	47^C^	Gln61Arg (c.182A>G)	WT
	48^C^	WT	WT
	49^C^	WT	WT
	50^O^,^C^	WT	WT
	51^O^,^V^,^C^	WT	WT
	52^V^,^C^	WT	WT
	53^O^,^C^	Gln61Arg (c.182A>G)	WT
	54^O^,^V^,^C^	WT	WT
	55^V^	WT	WT
	56^V^	WT	WT

O – oncocytic

V – vascular invasion

C – capsular invasion

### Predictive score for FTC diagnostics based on combined gene expression level changes

**Figure 1:**
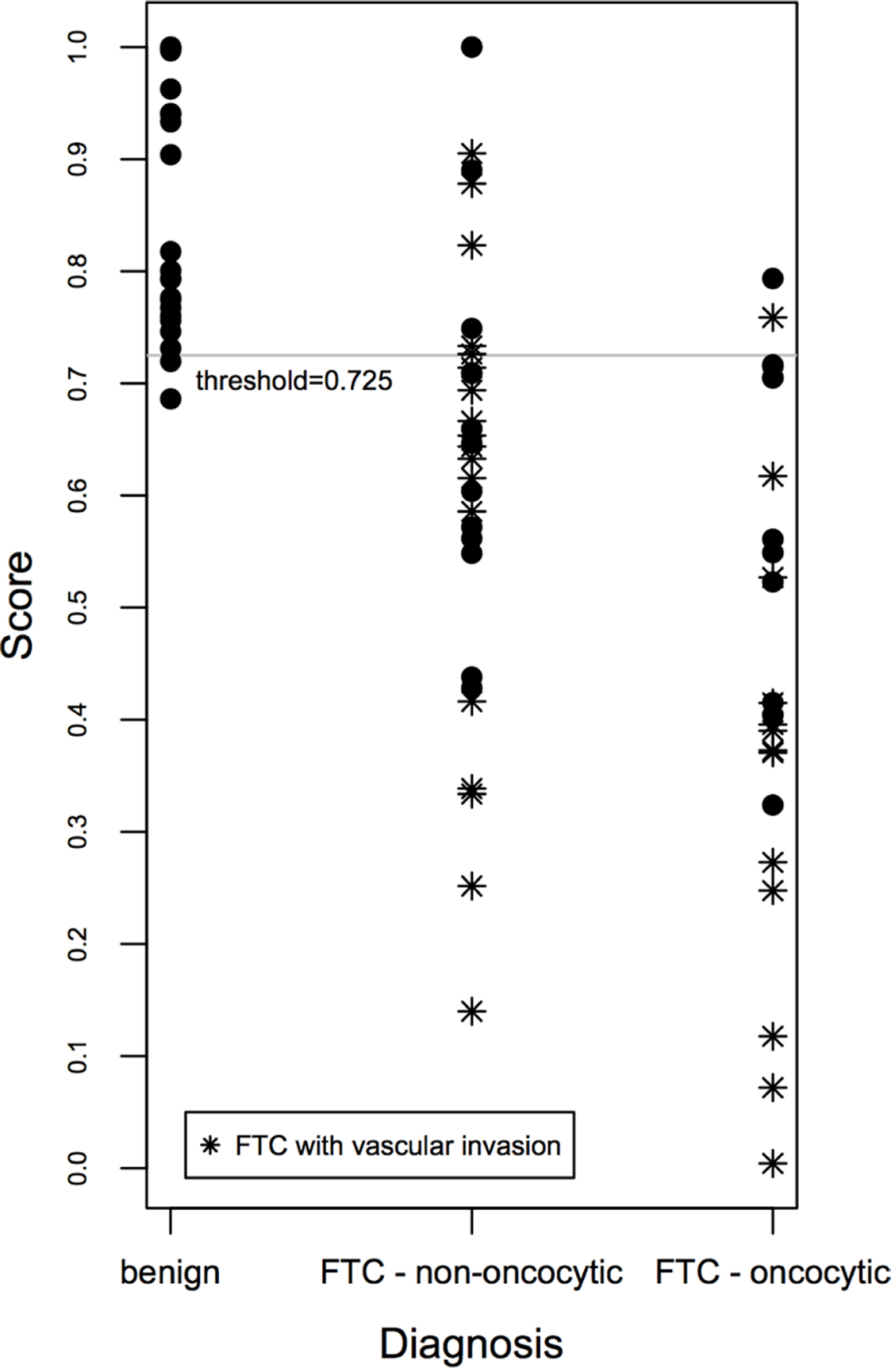
Scatter plot of the gene expression-based predictive score correlated with FTC aggressiveness The gene expression-based score for benign and FTC samples was calculated based on joint expression levels of *CHEK1, c-KIT, SLC26A4, TG* and *TPO* transcripts. Thus obtained score values were plotted for three clusters: benign, non-oncocytic FTC and oncocytic FTC, and allowed for a clear distinction between these groups.

In an attempt to correlate the degree of expression level changes of *CHEK1, c-KIT, SLC26A4, TG* and *TPO* with the histological diagnosis, we aimed at establishing a gene expression-based predictive score, taking into account the collective biomarker changes [12, 13]. A final predictive score was established for each biological sample, based on the expression levels of five distinctive genes (*CHEK1, c-KIT, SLC26A4, TG* and *TPO),* which exhibit stable changes in FTC and correlate among each other, using the Linear Prediction Score (LPS; for details see Supplementary Methods; [13]). To test the performance of the score, a receiver operating characteristic (ROC) analysis was performed, and ROC curves were established (see Supplementary Figure S1A and Supplementary Methods). Our results indicate that at a threshold of 0.725, based on empirical curve analysis (Supplementary Figure S1A, Supplementary Tables S6, S7), our predictive score discriminates FTC from benign cases with 96% sensitivity and 82% specificity. Of note, such discrimination was more sensitive for the oncocytic FTC cases compared to their non-oncocytic counterparts (significantly more false negatives for non-oncocytic than for oncocytic FTC, see Figure 1). Moreover, FTCs with vascular invasion exhibited the highest scores, if compared with their counterparts that do not bear vascular invasion (Figure 1).

In spite of the relatively high fold changes in FTC, *CHEK1* exhibited weak correlations with the rest of the identified biomarkers. To identify a cluster of biomarkers, which will give the most reliable predictive score, we tested predictive scores based on the combinations of *c-KIT, SLC26A4, TG, TPO* and *SLC26A4, TG, TPO* (Supplementary Figures S1B–S1C and S2A–S2B) that gave slightly weaker specificity (77%) or sensitivity (95%), respectively. Finally, a predictive score based on the combination of *BCL2, CHEK1, CRY2, KDR, c-KIT, PER2, SLC26A4, TG,* and *TPO* was established (Supplementary Figures S1D and S2C). It allows for the discrimination of FTC from benign with 97% sensitivity and 78% specificity. In agreement with the first predictive score (Figure 1), specificity of the predictive score was higher for the oncocytic subgroup compared to the non-oncocytic subgroup, and the highest for FTCs bearing vascular invasion. We thus conclude that the predictive score based on the combination of *CHEK1, c-KIT, SLC26A4, TG* and *TPO* allows for the most accurate prediction of FTC diagnosis (Figure 1). Although these results strongly suggest a predictive value for FTC diagnosis based on the combined assessment of 5 gene expression level changes, follow-up studies with a higher number of samples will be required to estimate the here proposed score validity.

In accordance with the gene expression-based score previously established by us for PTC, we employed the *BRAF* mutation status as an additional parameter for establishing the correlation with postoperative clinical evaluation [8]. However, due to the low frequency of the *N-RAS61* and *H-RAS61* mutation in our FTC cohort (8.9% and 1.8% respectively; Table 4), and lack of correlation between the mutation frequency and disease progression, the *RAS* mutational status was not taken into account for the predictive score established for FTC.

## DISCUSSION

### Altered transcript expression in human FTC: identification of new and confirmation of previously reported potential biomarkers by NanoString analysis

NanoString nCounter^TM^, a color-coded probe-based method, represents a highly sensitive approach for the quantification of gene expression. Based on direct probe hybridization, it allows for the collective assessment of a large number of transcripts within the same sample, including high precision analyses of FFPE samples, as previously reported by us and others [8, 14, 15].

Employing NanoString analysis, we report for the first time a strong upregulation of the essential cell cycle component *CHEK1* in FTC samples (Table 1). Alterations in *CHEK1* levels have been previously described by us in PTC (Table 5, [8]), and in a number of non-thyroid malignancies by other groups [16, 17]. A recent report reveals that *CHEK2* (but not *CHEK1*) levels are altered in PDTC and ATC [18]. In addition, transcript levels of the solute carrier (SLC) family members *SLC26A4* (encoding for pendrin) and *SLC5A5* were significantly downregulated in samples without consideration of FTC type, and in oncocytic FTC, respectively (Table 1). Our recent study revealed that *SLC26A4* has a tendency for downregulation in human PTC [8]. Interestingly, the *SLC26A4* gene methylation pattern in benign adenoma was altered in thyroid carcinoma, with methylation levels being inversely correlated to the gene expression levels, suggesting that such epigenetic changes might represent a new mechanism in altering *SLC26A4* gene function during thyroid carcinoma tumorigenesis [19]. *SLC5A5* was previously reported to be downregulated in thyroid carcinomas by us and others [8, 20]. Of note, pendrin was suggested to be a downstream target of the TTF-1/Nkx-2.1 homeodomain transcription factor in differentiated thyroid cells [21]. In good agreement with previous work [22], our current analysis reveals that thyroglobulin (*TG*) was significantly downregulated in FTC (Table 1). Assuming that thyroid tissue is de-differentiating upon carcinoma development, this might be a plausible mechanism by which *SLC26A4* and *TG* are downregulated in FTC. To further explore this link, it might be interesting to assess the expression of *TTF1* and *Nkx2.1* in the same human carcinoma samples in the future. With regard to the strong downregulation of *c-KIT* observed by us in FTC (Table 1), to the best of our knowledge such downregulation has not been previously associated with human FTC, while a similar tendency has been previously reported in PTC by us and others [8]. Finally, in an agreement with the previously established role of thyroid peroxidase (*TPO*) in oncogenic transformation in general, and its association with human thyroid carcinomas [23, 24], our analysis has shown a strong downregulation of this transcript in all FTC samples (Table 1).

**Table 5:** Comparative analysis of altered transcript expression in FTC, PTC* and PDTC samples

Gene	all FTC	oncocytic FTC with vascular invasion	all PTC*	more aggressive* PTC	all PDTC	oncocytic PDTC
*BCL2*	−1.48	−2.01	−3.17	−3.24	−2.63	−5.37
*CHEK1*	9.06	13.40	2.97	3.01	25.14	33.02
*CRY2*	−1.32	−1.71	−1.92	−2.02	−2.07	−3.38
*c-KIT*	−5.06	−10.13	−10.80	−12.21	−9.98	−43.36
*DIO2*	−1.61	−1.76	−3.61	−3.65	−3.10	−4.87
*FZD1*	−1.17	−2.23	NA	NA	−2.68	−5.53
*KDR*	−1.48	−1.73	NA	NA	−3.68	−5.90
*PER2*	−1.62	−2.90	−1.55	−1.63	−3.06	−6.46
*SLC26A4*	−2.19	−3.87	NS	NS	−17.79	−36.62
*TG*	−2.43	−5.42	−3.85	−4.23	−10.60	−37.29
*TPO*	−3.52	−5.42	−28.31**	−23.22**	−28.68	−62.72

NS - non significant.

NA - not assessed.

* Based on the results previously published by us [8].

** Highly variable expression.

In summary, our study reveals for the first time that transcript levels of *CHEK1* are strongly upregulated in human FTCs. Moreover, it further confirms downregulation of *SLC26A4, SLC5A5, c-KIT, TG*, and *TPO* in the same FTC samples, in good agreement with previous publications.

### Cell cycle regulators and core-clock components in human thyroid carcinomas

There is growing evidence on the importance of biological rhythms in the pathophysiology and treatment of cancer [25–28]. Recent findings have revealed that the circadian clock and cell cycle might be linked [29–32]. Here, we show for the first time a downregulation of *PER2* core-clock transcript levels in oncocytic FTC, and PDTC cases (Table 1–2). Of note, *PER2* has been previously demonstrated to play a key role as tumor suppressor, by regulating DNA damage responsive pathways [33]. The levels of another clock transcript, *CRY2,* were significantly downregulated in PDTC, and even further downregulated in oncocytic PDTC (Table 2), in agreement with our previous study, demonstrating downregulation of *CRY2* in PTC (Table 5 [8]). The alterations in expression levels of *PER2* and *CRY2* described here in PDTCs are in line with the results of our previous study, demonstrating that the molecular characteristics of the human thyroid clock are altered in primary cultured thyrocytes derived from PDTC biopsies [34]. Furthermore, a key cell cycle regulator *CHEK1* exhibited significant alterations in all groups of malignancies, with increasing fold changes from FTC to PDTC (Table 5). Additional cell cycle regulator *CDKN1B* was significantly downregulated in PDTCs (Table 2). Finally, we demonstrate that the apoptosis related gene *BCL2,* previously reported to be associated with a number of malignancies by other groups [16, 17], exhibits a downregulation in FTC, PTC and PDTC, with a progressive increase in fold change associated with malignancy progression (Table 5, [8]).

Taken together, these data suggest a correlation between the transcriptional changes in the levels of the circadian clock, the cell cycle key components, and the increasing the risk for oncogenic transformation and progression. Providing further insights into the molecular mechanisms that underlie the alterations in key components of the core-clock, cell cycle and apoptosis, and their roles in thyroid malignancy progression, might be of great scientific and clinical interest.

### Correlation between the molecular biomarker alterations and the clinical progression of human thyroid carcinomas

Strikingly, the pattern of molecular biomarkers identified by our analyses was strongly associated with the clinical diagnostics of the FTC and PDTC subgroups (Tables 1–2). Both oncocytic FTC and PDTC groups exhibited a higher number of altered genes compared to their non-oncocytic counterparts. For instance, a key component of *WNT* signaling, *FZD1*, whose downregulation might be associated with increased growth and invasiveness of FTCs [35], was significantly decreased in oncocytic FTC and PDTC only, while it stayed unchanged in non-oncocytic samples (Tables 1–2). In addition, transcripts, which were altered in both oncocytic and non-oncocytic subgroups, exhibited consistently higher fold-changes in the oncocytic group versus non-oncocytic counterparts (compare subgroups 4–6 to 2–3 in Table 1 and subgroups 3 and 2 at Table 2). These data further support the hypothesis that oncocytic and non-oncocytic variants of human thyroid carcinomas might bear distinct molecular pattern [36]. Additionally, FTC with vascular invasion exhibited more pronounced changes of molecular markers, if compared to their counterparts without vascular invasion (Table 1), further suggesting that vascular invasion represents a hallmark of malignancy, accompanied by dramatic changes in the molecular pattern [36].

Of note, the comparative investigation of gene expression levels assessed by NanoString analyses in three major clinical groups of human thyroid carcinomas (FTC, PTC and PDTC [8]), reveals that alterations levels of several transcripts might be gradually increasing in conjunction with tumor progression (Table 5). Such tendency was observed for *BCL2, CRY2, c-KIT, DIO2, FZD1, KDR, PER2, SLC26A4, TG* and *TPO,* (Table 5). For *CHEK1*, however, alteration levels in PTC were lower than those observed for FTC, which might be attributed to the relatively small number of cohorts analyzed in both studies.

### Towards a reliable correlation coefficient for the diagnosis of FTC: a gene expression-based predictive score

Correlation analysis of the most promising biomarkers for FTC (*CHEK1, c-KIT, SLC26A4, TG* and *TPO)* allowed for the establishment of a predictive score that discriminates between benign and FTC samples with 96% sensitivity and 82% specificity at a threshold of 0.725 (Figure 1). While this score was moderately reliable for the non-oncocytic subgroup of FTC (Figure 1), in case of oncocytic FTC only two false-negatives were observed (Figure 1). The most reliable prediction was provided for oncocytic cases with vascular invasion, based on the subset of samples analyzed in our work (Figure 1). Importantly, our predictive score is only indicative at this point and demands rigorous confirmation in subsequent follow-up studies.

Assessment of preoperative biomarkers for thyroid carcinomas through microRNA screening [37], proteome, and lipidome analyses [38, 39] have recently proven to be highly promising strategies. Thus, integrative approaches including the here established predictive score based on the combined alterations of several molecular biomarker levels, possibly in combination with biomarkers assessed by microRNA, proteomic and lipidomic profiling, might encompass a great potential towards increasing the reliability of the preoperative diagnostics for thyroid carcinomas.

## MATERIALS AND METHODS

### Study participants and thyroid tissue sampling

FFPE samples from benign, FTC and PDTC human thyroid nodules were obtained from the archive of the Pathology Department, Geneva University Hospital. Donor characteristics are summarized in Supplementary Tables S1–S3. Malignant tumors were classified by histopathological analysis according to the World Health Organization Classification of Thyroid Tumors [40] and staged according to the AJCC Cancer Staging Manual 7th ed (see Supplementary Data for more details on the diagnostics). In addition, the diagnostics of PDTC cases was made using the Turin criteria [41]. Written informed consent was obtained from each patient and the study protocol was approved by the local Ethics Committee (CER 11-014).

### RNA extraction from FFPE samples

RNA was extracted using the High Pure miRNA isolation kit (Roche) according to the manufacturer's instruction, as previously described by us in details [8].

### Gene expression quantification using multiplexed, color-coded probe pairs (NanoString nCounterTM)

53 candidate genes were selected for analysis, based on our own previous studies [8, 34], and on literature search. Several transcripts, previously demonstrated to exhibit strong expression level changes in the FTC and PDTC, such as TIMP1, c-MET and c-KIT [20, 42], were included for the correlation analysis. Probes were designed and synthesized by NanoString nCounterTM technologies. 22 genes out of 53 overlapping between the three independent NanoString experiments (codesets), exhibited significant alterations in thyroid carcinoma (Supplementary Table S4) and were therefore used for subsequent analyses. 200–400 ng of total RNA, extracted from FFPE samples, were hybridized with multiplexed NanoString probes, as described in [9], from 3 independent NanoString experiments (codesets 1, 2 and 3). Background correction, codeset calibration, and statistical analysis were performed as described in Supplementary Methods.

### RAS mutation analysis

For the analysis of *N-RAS61* and *H-RAS61* mutations, 3 μm thick FFPE tissue sections were deparaffinized in xylol, proteins were digested over night at 56°C and DNA was subsequently processed on the QIAcube using the QIAamp DNA FFPE Tissue Kit (QIAGEN). PCR was performed as previously described [43] using the following primers: *HRAS*61 forward 5′-TGTCCTCCTGCAGGATTC-3′ and reverse 5′-GTACTGGTGGATGTCCTC-3′, *NRAS*61 forward 5′-CACCCCCAGGATTCTTACAG-3′ and reverse 5′-TCCGCAAATGACTTGCTATT-3′. PCR products were separated on agarose gel, purified using the peqGold gel extraction kit (Peqlab), sequenced and analyzed on a capillary automatic sequencer (Applied Biosystems 96-capillary 3730xl).

## SUPPLEMENTARY MATERIALS FIGURES AND TABLES



## Abbreviations

ATC: Anaplastic Thyroid Carcinoma
BCL2: B-Cell CLL/Lymphoma 2
BMAL1: Brain and muscle ARNT-like protein 1
CHEK1: Checkpoint Kinase 1
c-KIT: V-Kit Hardy-Zuckerman 4 Feline Sarcoma; Viral Oncogene Homolog
CRY2: Cryptochrome 2
DIO2: Deiodinase, Iodothyronine, Type II
FDR: False Discovery Rate
FTC: Follicular Thyroid Carcinoma
FFPE: Formalin-fixed Paraffin-embedded
FNA: Fine Needle Aspiration
FZD1: Frizzled Class Receptor 1
HRAS: Harvey Rat Sarcoma Viral Oncogene; Homolog
KDR: Kinase Insert Domain Receptor
Nkx-2.1: NK2 Homobox 1
NRAS: Neuroblastoma RAS Viral Oncogene; Homolog
PAX 8: Paired box 8
PER2: Period 2
PDTC: Poorly Differentiated Thyroid Carcinoma
PPARγ: Peroxisome Proliferator-Activated Receptor; Gamma
PTC: Papillary Thyroid Carcinoma
ROC: Receiver Operating Characteristic
SLC26A4: Solute Carrier Family 26 (Anion, Exchanger), Member 4
SLC5A5: Solute Carrier Family 5 (Sodium/Iodide, Cotransporter), Member 5
TTF1: Transcription Termination Factor, RNA; Polymerase I
TG: Thyroglobulin
TIMP1: Tissue Inhibitor of Metalloproteinase 1
TPO: Thyroid Peroxidase
WEE1: G2 Checkpoint Kinase.
