# Supplementary material for: Identification of CHEK1, SLC26A4, c-KIT, TPO and TG as new biomarkers for human follicular thyroid carcinoma
Source: Oncotarget. 2016 Jun 18;7(29):45776–88. doi: 10.18632/oncotarget.10166 (PMC5216760; doi:10.18632/oncotarget.10166)
Supplement: Supplementary file 1 [file oncotarget-07-45776-s001.pdf]

Identification of CHEK1, SLC26A4, c-KIT, TPO and TG as new biomarkers for human follicular thyroid carcinoma

Supplementary Materials

SUPPLEMENTARY METHODS

Diagnostics of FTC and PDTC nodules

Tumors were classified histologically according to the current World Health Organization criteria [1]. A well-circumscribed or encapsulated, cytologically bland follicular lesion was considered FA in the absence of capsular and vascular invasion. A cytologically bland, encapsulated follicular lesion was considered FTC if there was transcapsular penetration or vascular invasion. An invasive neoplasm comprised of follicular cells (confirmed by positive thyroglobulin and negative calcitonin immunostaining) growing in trabeculae or solid sheets, without nuclear features of PTC, and with one of the following (convoluted nuclei, mitoses ≥ 3 per 10 high power fields, or tumor necrosis) was considered poorly differentiated thyroid carcinoma (PDTC), according to the Turin proposal [2, 3]. Tumors were defined as oncocytic when more than 70% of the tumor was composed of oncocytic cells (i.e., round to polygonal cells with abundant eosinophilic granular cytoplasm and round nuclei with prominent nucleoli).

Merge of the results obtained in three independent NanoString analyses

Background correction was made by subtracting the mean + 2 SD of the raw counts obtained with negative controls. Values < 1 were fixed to 1 to avoid negative values after log transformation (see [4] for details). Counts for target genes were then normalized with the geometric mean of four house-keeping genes (*ACTB, EEF1A1, HPRT1* and *RPL13A*) selected as the most stable using the geNorm algorithm [5].

Normalized expression values from the codesets 1 and 2 are adjusted to normalized expression values from the codeset 3.

Calibration samples have been tested on all codesets and are used for this adjustment.

Codeset 1 adjustment:

1. for each gene in common (×22), a calibration factor (CF) was calculated:

$$CF_{gene} = \frac{\text{mean of normalized\_expression \_value}_{\text{gene sample code-set 1}}}{\text{mean of normalized\_expression \_value}_{\text{gene sample code-set 3}}}$$

2. then:

$$\text{Adjusted\_normalized\_expression\_value}_{\text{sample code-set 1/gene}} = \text{normalized\_expression\_value}_{\text{sample code-set 1/gene}} \times CF_{\text{gene}}$$

Codeset 2 is adjusted to the same way.

Statistical analysis

To assess differences in gene-expression values between the different groups, ANOVA tests with contrasts (significant *P*-values < 0.05) were performed using Partek Genomics Suite (http://www.partek.com), as previously described by us [4]. *P*-values were corrected for multiple testing by use of the false-discovery rate (FDR) method of Benjamini and Hochberg [6]. A conservative significance threshold of 5% FDR associated with fold change value of 2 or more was applied. Merge of the results obtained in three independent NanoString runs was performed as described in Supplementary methods and previously in [4]. Pair-wise Pearson correlation analysis was applied to test the correlation between gene expression data obtained by NanoString. Correlation strength was interpreted as proposed by Evans [5].

Gene score data analysis

Predictive score for each biological sample was based on the combined expression levels of chosen genes exhibiting stable changes in FTCs compared to benign samples. To ensure the reproducibility of our model, we equally divided 22 benign (Table 1) and 56 FTC (Tables 1–2) samples into training and validation sets by computer-based randomization (test on 1000 random selections [7, 8]). The training set was used to create and optimize the gene-expression score, while the validation set allowed testing the model and evaluating its significance. Using the training set and t-test analysis we

chose the genes that were differentially expressed in the benign and FTC samples (see Supplementary Table 6 for gene selection):

1. *CHEK1, c-KIT, SLC26A4, TG* and *TPO* genes present high fold-change expression (*fold*-change > 2) and high significance (*t-test P-value* < 0.05) in at least 700 selections out of 1000 tested.
2. *c-KIT, SLC26A4, TG* and *TPO* genes present high fold-change expression (*fold*-change > 2) and high significance (*t-test P-value* < 0.05) in at least 700 selections out of 1000 tested.
3. *SLC26A4, TG* and *TPO* genes present high significance (*t-test P-value* < 0.05) in all tested selections.
4. *BCL2, CHEK1, CRY2, KDR, c-KIT, PER2, SLC26A4, TG* and *TPO* genes present high significance (*t-test P-value* < 0.05) in at least 700 selections out of 1000 tested.

To calculate FTC prediction score, first the expression levels of distinction genes were combined to calculate a Linear Predictor Score (LPS) [9] for each sample (X) in the training set:

$$LPS(X) = \sum_j a_j X_j$$

Where:

X_j –a gene expression value of gene j

a_j –a scaling factor, whose value depends on the degree, to which each gene discriminates in the subgroup. The scaling factors were chosen to be the t-statistics generated by a t-test for the difference in expression between FTC and benign subgroups.

To obtain the FTC prediction score comprised between 0 and 1, the following score transformation was performed:

$$Score\ (X) = (LPS(X) – \text{min of LPSs})/(\text{max of LPSs} – \text{min of LPSs})$$

A similar procedure of FTC prediction score calculation was used for validation set. The score values for all samples are shown in Supplementary Table 7.

To test the performance of the score, a receiver operating characteristic (ROC) analysis with calculation of the ROC area under the curve (AUC) statistic was performed [7, 8, 10]. The ROC curve (Supplementary Figure 1) gives sensitivity and specificity of a certain test in function of the cut off value: the better the diagnostic test, the nearer the curve reaches to the top left corner, indicating 100% sensitivity and specificity.

REFERENCES

1. The International Agency for Research on Cancer (Author) RDE, R. Lloyd (Editor), P. Heitz (Editor), C. Eng (Editor) (2004). Pathology and Genetics of Tumours of Endocrine Organs (IARC WHO Classification of Tumours). Blue Book.
2. Volante M, Landolfi S, Chiusa L, Palestini N, Motta M, Codegone A, Torchio B, Papotti MG. Poorly differentiated carcinomas of the thyroid with trabecular, insular, and solid patterns: a clinicopathologic study of 183 patients. Cancer. 2004; 100:950–957.
3. Volante M, Collini P, Nikiforov YE, Sakamoto A, Kakudo K, Katoh R, Lloyd RV, LiVolsi VA, Papotti M, Sobrinho-Simoes M, Bussolati G, Rosai J. Poorly differentiated thyroid carcinoma: the Turin proposal for the use of uniform diagnostic criteria and an algorithmic diagnostic approach. Am J Surg Pathol. 2007; 31:1256–1264.
4. Chitikova Z, Pusztaszeri M, Makhlouf AM, Berczy M, Delucinge-Vivier C, Triponez F, Meyer P, Philippe J, Dibner C. Identification of new biomarkers for human papillary thyroid carcinoma employing NanoString analysis. Oncotarget. 2015; 6:10978–10993.
5. Vandesompele J, De Preter K, Pattyn F, Poppe B, Van Roy N, De Paepe A and Speleman F. Accurate normalization of real-time quantitative RT-PCR data by geometric averaging of multiple internal control genes. Genome Biol. 2002; 3:RESEARCH0034.
6. Hochberg Y and Benjamini Y. More powerful procedures for multiple significance testing. Stat Med. 1990; 9:811–818.
7. Robin X, Turck N, Hainard A, Tiberti N, Lisacek F, Sanchez JC and Muller M. pROC: an open-source package for R and S+ to analyze and compare ROC curves. BMC Bioinformatics. 2011; 12:77.
8. Rosenberg S, Elashoff MR, Beineke P, Daniels SE, Wingrove JA, Tingley WG, Sager PT, Sehnert AJ, Yau M, Kraus WE, Newby LK, Schwartz RS, Voros S, et al. Multicenter validation of the diagnostic accuracy of a blood-based gene expression test for assessing obstructive coronary artery disease in nondiabetic patients. Ann Intern Med. 2010; 153:425–434.
9. Wright G, Tan B, Rosenwald A, Hurt EH, Wiestner A and Staudt LM. A gene expression-based method to diagnose clinically distinct subgroups of diffuse large B cell lymphoma. Proc Natl Acad Sci U S A. 2003; 100:9991–9996.
10. Brutsche MH, Joos L, Carlen Brutsche IE, Bissinger R, Tamm M, Custovic A and Woodcock A. Array-based diagnostic gene-expression score for atopy and asthma. J Allergy Clin Immunol. 2002; 109:271–273.

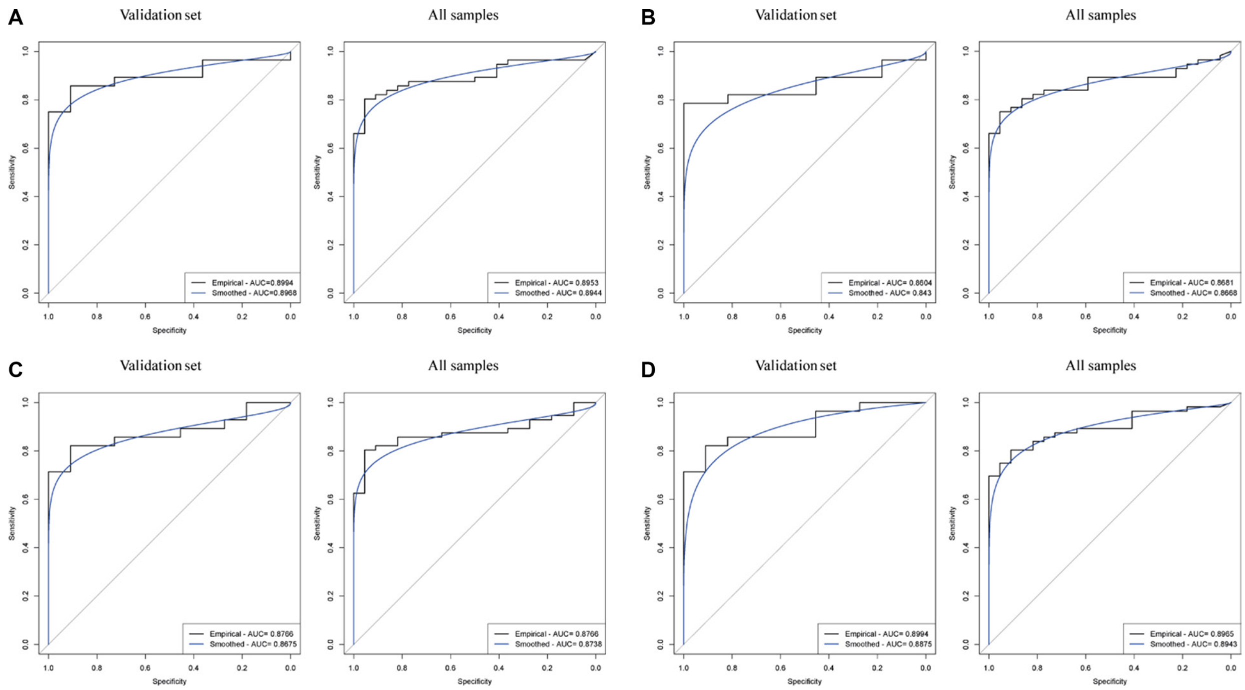

Supplementary Figure S1: ROC analysis. ROC analysis with calculation of the area under the curve (AUC) statistic was performed to test in validation set (left) and all samples (right), based on the following biomarker combinations: (**A**) *CHEK1, c-KIT, SLC26A4, TG, TPO:* AUC = 0.89; at the threshold of 0.725, the diagnostic score discriminates FTC from benign with 96% sensitivity and 82% specificity in the validation set (left) and all sample analysis (right); (**B**) *c-KIT, SLC26A4, TG, TPO:* AUC = 0.87; at the threshold of 0.833, the diagnostic score discriminates FTC from benign with 96% sensitivity and 77% specificity in the validation set (left) and all sample analysis (right); (**C**) *SLC26A4, TG, TPO:* AUC = 0.88; at the threshold of 0.832, the diagnostic score discriminates FTC from benign with 95% sensitivity and 82% specificity in the validation set (left) and all sample analysis (right); (**D**) *BCL2, CHEK1, CRY2, KDR, c-KIT, PER2, SLC26A4, TG, TPO:* AUC = 0.90; at the threshold of 0.701, the diagnostic score discriminates FTC from benign with 97% sensitivity and 78% specificity in the validation set (left) and all sample analysis (right). Binormal smoothing iteration was performed to show continuous ROC curves in all cases.

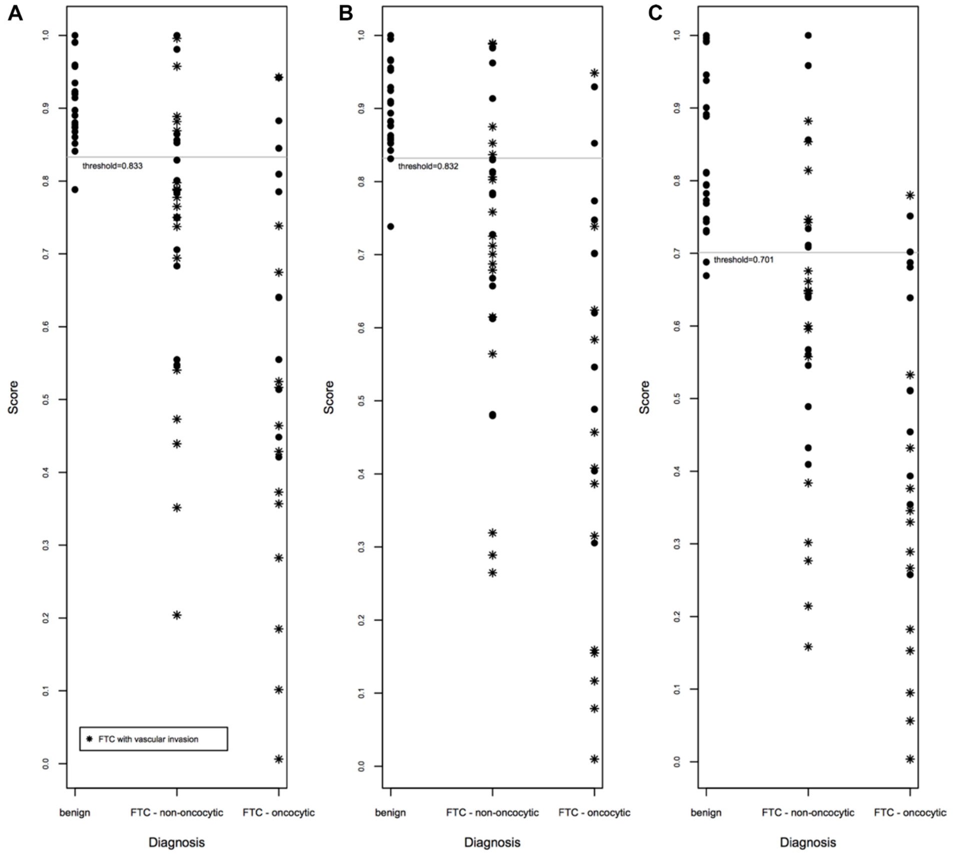

Supplementary Figure S2: Scatter plot of gene expression-based predictive scores correlated to FTC biological aggressiveness. The gene expression-based score for benign and FTC samples was calculated based on joint expression levels of (**A**) *C-KIT, SLC26A4, TG, TPO*; (**B**) *SLC26A4, TG, TPO*; and (**C**) *BCL2, CHEK1, CRY2, KDR, C-KIT, PER2, SLC26A4, TG, TPO*.

Supplementary Table S1: Donors' characteristics and diagnosis: healthy thyroid tissue and benign nodules

Healthy tissue				Benign nodules			
Case	Sex	Age, years	Size (cm)	Case	Sex	Age, years	Size (cm)
1	F	58	N.A	1	M	67	3.8
2	M	83	N.A	2	F	53	3.5
3	F	28	N.A	3	M	30	3.5
4	F	32	N.A	4	F	36	2.5
5	F	47	N.A	5	F	30	3
6	F	38	N.A	6	F	37	3.5
7	M	39	N.A	7	F	54	4
8	F	48	N.A	8	M	74	3
9	F	47	N.A	9	F	74	4
10	F	42	N.A	10	F	66	2.9
11	F	64	N.A	11	F	54	3
12	F	48	N.A	12	F	51	1.3
				13	F	44	2.8
				14	F	47	2.9
				15	F	34	4
				16	F	43	5
				17	F	53	5.5
				18	F	42	3
				19	M	48	3.8
				20	F	83	1.8
				21	F	63	2.7
				22	M	43	2.5

N.A – Not applicable.

Supplementary Table S2: Donors' characteristics and diagnosis: FTC nodules

Case	Sex	Age, years	Size (cm)	Case	Sex	Age, years	Size (cm)
1[V,C]	M	54	1.2	29[O,C]	F	61	2.7
2[O,V,C]	M	47	2.3	30[O,V,C]	M	46	5.5
3[O,C]	F	23	4	31[O,V,C]	M	35	4.2
4[O,V,C]	F	45	2.2	32[O,C]	F	75	1.6
5[V,C]	F	32	3.5	33[O,V,C]	F	76	3.6
6[C]	F	43	2,3	34[O,V,C]	F	25	3
7[V,C]	M	55	2	35[O,V,C]	F	59	2.5
8[V,C]	M	79	2.6	36[O,C]	F	38	5
9[C]	F	51	2.4	37[O,C]	F	61	3
10[C]	F	48	3.5	38[O,C]	F	64	2
11[O,V]	M	38	2.9	39[O,V,C]	M	48	3.4
12[V]	F	61	6.5	40[V]	F	46	2
13[V]	M	52	3	41[C]	F	62	6.5
14[C]	F	53	1.4	42[C]	F	53	2.1
15[O,V,C]	F	56	3.5	43[V,C]	F	32	4.1
16[C]	F	52	1.7	44[V,C]	F	35	3
17[C]	F	61	1.5	45[C]	F	54	4.2
18[V]	F	71	2.5	46[C]	F	47	3
19[C]	F	34	1.9	47[C]	F	62	2.7
20[V,C]	F	40	4.2	48[C]	F	38	6.5
21[V]	F	30	2.5	49[C]	F	23	1.8
22[V]	F	40	2	50[O,C]	F	40	3.8
23[V]	F	59	2.8	51[O,V,C]	F	52	2.5
24[O,C]	F	69	1.7	52[V,C]	F	48	2.2
25[O,C]	F	33	1.9	53[O,C]	F	41	5
26[V]	F	52	1.5	54[O,V,C]	F	64	2.9
27[O,C]	F	70	1.5	55[V]	F	31	2.5
28[O,V,C]	M	80	6.5	56[V]	F	30	3

O – oncocytic; V – vascular invasion; C – capsular invasion.

Supplementary Table S3: Donors' characteristics and diagnosis: PDTC nodules

Case	Sex	Age, years	Size (cm)
$1^{V,C}$	F	47	4.5
$2^{V,C}$	F	40	6.3
3	M	68	8.3
$4^{V,C}$	F	69	10
$5^{V,C}$	F	34	6
$6^{V,C}$	F	20	2.5
$7^{O,V,C}$	M	80	7
$8^{V,C}$	F	75	ND
$9^{V,C}$	F	81	4.8
$10^{V,C}$	M	47	3.5
$11^{O,V,C}$	M	59	6
$12^{O,V,C}$	M	79	ND
$13^{V,C}$	F	70	5.3
14°	M	85	6
15°	M	58	4.6
16°	F	78	1.5
17	M	79	8
18	M	87	5.5
19°	M	80	6.5
20	F	64	3.3
21	F	68	8
22°	M	56	ND
23°	F	67	ND
24°	F	74	4
25^{C}	F	70	3

O – oncocytic; V – vascular invasion; C – capsular invasion;
ND – Non-defined (samples obtained from the lymph node metastasis).

Supplementary Table S4: Nanostring probes design

Gene	Accession	Target Region	Gene	Accession	Target Region
ACTB[h]	NM_001101.2	1011–1110	*MET*	NM_000245.2	406–505
ARNTL	NM_001030272.1	841–940	*PER1*	NM_002616.2	4366–4465
BCL2	NM_000657.2	6–105	*PER2*	NM_022817.2	986–1085
CDKN1B	NM_004064.2	366–465	*PPARG*	NM_015869.3	1036–1135
CHEK1	NM_001114121.1	2226–2325	*PTGS2*	NM_000963.1	496–595
CRY2	NM_001127457.1	3326–3425	*RPL13A*[h]	NM_012423.2	721–820
DDIT3	NM_004083.4	41–140	*SFRP1*	NM_003012.3	3321–3420
DIO2	NM_013989.3	5076–5175	*SLC26A4*	NM_000441.1	1711–1810
EEF1A1[h]	NM_001402.5	791–890	*SLC5A5*	NM_000453.2	3459–3558
FZD1	NM_003505.1	2431–2530	*TG*	NM_003235.4	6499–6598
HPRT1[h]	NM_000194.1	241–340	*TIMP1*	NM_003254.2	330–429
KDR	NM_002253.2	1421–1520	*TPO*	NM_175719.3	297–396
c-KIT	NM_000222.1	6–105	*WEE1*	NM_003390.3	1226–1325

[h]House-keeping genes.

Supplementary Table S5: Twenty-two transcripts assessed by NanoString analysis exhibit comparable levels in healthy thyroid and benign thyroid nodule samples

Gene	*P*-value (benign vs. healthy)	*P*-value with FDR (benign vs. healthy)	Fold-Change (benign vs. healthy)	Total number of samples benign / healthy	Number of samples with expression value > 50 (linear scale) – benign/ healthy
ARNTL	0.0433	0.6274	1.59	22/12	22/12
BCL2	0.7106	0.9303	–1.08	22/12	22/12
CDKN1B	0.1213	0.6672	–2.57	22/12	22/12
CHEK1	0.0855	0.6274	2.99	22/12	10/9
CRY2	0.5779	0.9303	–1.09	22/12	22/12
DDIT3	0.2079	0.7622	–1.39	22/12	22/12
DIO2	0.4471	0.9303	–1.29	22/12	22/12
FZD1	0.6245	0.9303	1.15	22/12	22/12
KDR	0.5443	0.9303	–1.13	22/12	22/12
KIT	0.9598	0.9774	–1.04	22/12	22/12
MET	0.6877	0.9303	1.09	22/12	22/12
PER1	0.8108	0.9774	–1.06	22/12	22/12
PER2	0.9214	0.9774	–1.02	22/12	22/12
PPARG	0.5705	0.9303	1.41	22/12	21/12
PTGS2	0.0789	0.6274	4.24	22/12	17/11
SFRP1	0.7189	0.9303	–1.51	22/12	15/12
SLC26A4	0.9774	0.9774	1.02	22/12	22/12
SLC5A5	0.1858	0.7622	3.39	22/12	19/12
TG	0.4241	0.9303	–1.46	22/12	22/12
TIMP1	0.3217	0.8846	1.35	22/12	22/12
TPO	0.3139	0.8846	–1.91	22/12	22/12
WEE1	0.9147	0.9774	1.02	22/12	22/12

Significant change was defined at FDR *p*-value < 0.05 and fold-change > 2, with no transcripts fitting these criteria.

Supplementary Table S6: Gene selection

Gene	number of times with *p*-value < 0.05 out 1000	number of times with fold-change > 2 out 1000	number of times with significant *p*-value and high fold-change out 1000
ARNTL	9	0	0
BCL2	971	0	0
CDKN1B	10	194	10
CHEK1	831	998	831
CRY2	875	0	0
DDIT3	24	0	0
DIO2	360	153	149
FZD1	25	0	0
KDR	865	2	2
c-KIT	999	999	999
MET	225	0	0
PER1	36	0	0
PER2	913	18	18
PPARG	8	25	7
PTGS2	15	425	15
SFRP1	24	634	24
SLC26A4	1,000	729	729
SLC5A5	110	767	110
TG	1,000	943	943
TIMP1	6	1	1
TPO	1,000	1,000	1,000
WEE1	214	0	0

Supplementary Table S7: Diagnostic subgroup definition using gene expression-based score. See Supplementary_Table_S7
